# Effects of seat pan and pelvis angles on the occupant response in a reclined position during a frontal crash

**DOI:** 10.1371/journal.pone.0257292

**Published:** 2021-09-20

**Authors:** Cyrille Grébonval, Xavier Trosseille, Philippe Petit, Xuguang Wang, Philippe Beillas

**Affiliations:** 1 Université Lyon, Université Claude Bernard Lyon 1, Université Gustave Eiffel, IFSTTAR, LBMC UMR_T 9406, Lyon, France; 2 Laboratory of Accidentology and Biomechanics, LAB PSA Peugeot-Citroen Renault, Nanterre, France; Virginia Tech, UNITED STATES

## Abstract

Current highly automated vehicle concepts include reclined seat layouts that could allow occupants to relax during the drive. The main objective of this study was to investigate the effects of seat pan and pelvis angles on the kinematics and injury risk of a reclined occupant by numerical simulation of a frontal sled test. The occupant, represented by a detailed 50th percentile male human body model, was positioned on a semi-rigid seat. Three seat pan angles (5, 15, and 25 degrees from the horizontal) were used, all with a seatback angle of 40 degrees from the vertical. Three pelvis angles (60, 70, and 80 degrees from the vertical), representing a nominal and two relaxed sitting positions, were used for each seat pan angle. The model was restrained using a pre-inflated airbag and a three-point seatbelt equipped with a pretensioner and a load limiter before being subjected to two frontal crash pulses. Both model kinematic response and predicted injury risk were affected by the seat pan and the pelvis angles in a reclined seatback position. Submarining occurrence and injury risk increased with lower seat pan angle, higher pelvis angle, and acceleration pulse severity. In some cases (in particular for a 15 degrees seat pan), a small variation in seat pan or pelvis angle resulted in large differences in terms of kinematics and predicted injury. This study highlights the potential effects of the seat pan and pelvis angles for reclined occupant protection. These parameters should be assessed experimentally with volunteers to determine which combinations are most likely to be adopted for comfort and with post mortem human surrogates to confirm their significance during impact and to provide data for model validation. The sled and restraint models used in this study are provided under an open-source license to facilitate further comparisons.

## Introduction

In highly automated vehicles (HAVs), i.e. level 4 or above, the occupant is no longer driving. This may allow new activities, such as conversing, working, relaxing, or sleeping [1]. New vehicle interiors will likely be needed to accommodate these activities and reclined seats were found desirable in several studies [2]. However, based on accident data, Dissanaike et al. [3] observed that reclined positions improperly used in current vehicles were associated with increased mortality.

Current restraint systems are designed and evaluated using Anthropomorphic Test Dummy (ATD) in a nominal seating position (i.e. 25 degrees manikin torso angle) [4]. Recent studies used human body models (HBM) to investigate the effects of reclined seating on the occupant kinematics and interactions with current restraint systems. All suggest that reclined seating increases the submarining risk compared to the nominal position. Besides, the submarining occurrence increases with more reclined seatback [5], smaller occupants (e.g. 5th percentile female model) [6], large lap belt angles [5]. Gepner et al. [7] also found that submarining occurrence is dependent on the HBM used. While these studies described in detail the occupant kinematics and the submarining behavior, they did not analyze the injury risk predicted for various body regions. The use of simplified models with limited injury prediction capability may have contributed to this (e.g. simplified version of the Global Human body Model Consortium 50^th^ percentile male model used in Boyle et al. [5]). Also, the restraint environment differed between studies but did not include some of the most common safety measures (e.g. airbag).

Concerning the seating configurations, the seatback angle was the primary focus of these studies. While the pelvis-seating [8] and seat pan angles may affect the kinematics and submarining risk in nominal seating configurations, these were fixed in previous studies (i.e. seat pan set to 15 degrees). However, for a more reclined position, occupants may prefer a higher seat pan angle for comfort (e.g. Theodorakos et al. [9]). Additionally, although a high pelvis angle variability (standard deviation over 10 degrees) was observed between participants for both upright [10] and reclined [11] positions, past simulation studies used only one pelvis angle by seat configuration (e.g. 60 degrees for the 45° Reclined Posture in Boyle et al. [5]).

Therefore, the objectives of the current study were to assess the occupant response in a reclined position for different seat pan and pelvis angles. A detailed HBM was used to simulate the occupant response in a frontal impact to allow investigating at the same time kinematics and injury predictions (using both injury criteria and local failure). Restraint conditions aiming to better represent current protection systems than in past simulation studies were also used, including a semi-rigid seat with an anti-submarining ramp, an airbag, and a three-point seat belt including pretensioners and load limiters. Also, in order to help with the reproducibility of the study and future comparisons with other models, a particular attention was paid to the documentation of the postural parameters and the sled environment.

## Material and methods

Occupant kinematics and restraint systems were simulated using finite element (FE) models of a sled and a HBM, the midsize male detailed occupant model from the Global Human Body Model Consortium (GHBMC M50-O V5.0). Rib and pelvis fractures were enabled. Simulations were performed using LS-DYNA (Version 971, R9.3.0, Livermore Software Technology).

### Environment model

**Fig 1:**
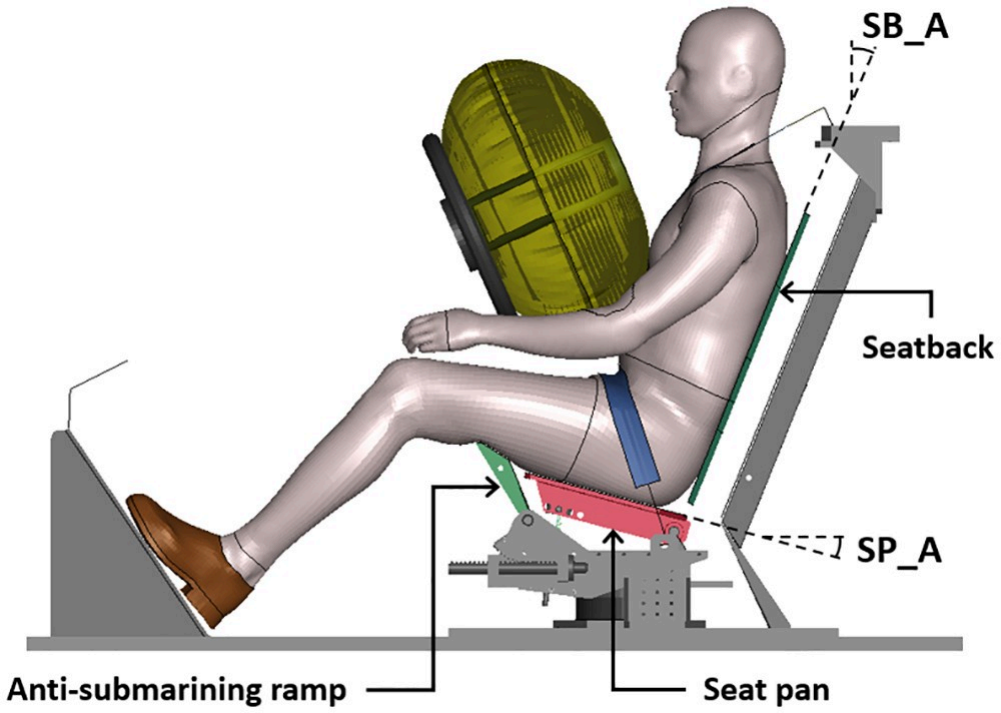
Environment model used (side view). SB_A: Seatback angle; SP_A: Seat pan angle.

The seat model corresponds to a physical seat called semi-rigid by their designer [12]. It aims to represent the behavior of real vehicle seats and can be configured to represent various characteristics in terms of stiffness or geometry [12]. It is composed of three adjustable articulated rigid plates: a seat pan, an anti-submarining ramp, and a seatback (Fig 1). The seat pan is a 380 mm width rigid plate articulated at its rear edge, using springs fixed under the front part of the plate. The anti-submarining ramp is positioned in front of the seat pan. The seat pan and the ramp can both rotate with a rotational stiffness adjusted using springs. This seat was previously developed to help with reproducibility in PMHS tests while providing a reasonable environment to study submarining and is currently used in several PMHS studies (e.g. Richardson et al. [13–16]). For the current study, the frontal seat configuration from Uriot et al. [12] was used. The seat pan and anti-submarining ramp were initially set to 15 and 32 degrees from the horizontal, respectively, and a seatback angle set to 22 degrees from the vertical [12].

The seat model was completed with a three-point seatbelt and a pre-inflated airbag as used in Trosseille et al. [17]. Shoulder and lap belts were both equipped with pretensioners, with time to fire set to 18 and 25ms, respectively [17]. The shoulder belt had a 3kN force limiter on the D-ring side, which is just below the peak force in Trosseille et al. [17] and Richardson et al. [15]. Based on simulation trials, a 30N load during 50ms was added at the beginning of the simulation to remove any belt slack and enhance the coupling with the belt.

A generic model of the airbag was derived from a medium-size European car (volume of 55 liters). No knee bolster was used as a typical knee bolster location would contact the lower extremities in reclined configurations with a high seat pan angle (preventing the positioning). A footrest was defined as it was compatible with all configurations and could limit submarining in reclined configurations [5].

**Fig 2:**
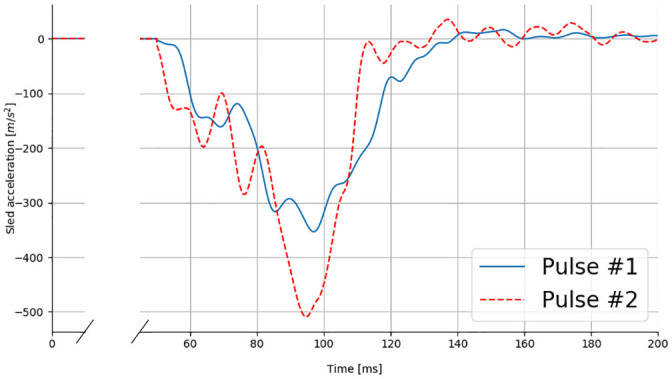
Sled acceleration pulses. The blue solid line and the red dashed line correspond to Pulse #1 and Pulse #2, respectively.

Two acceleration pulses were used (Fig 2). The first pulse used (Pulse #1) is a literature pulse: it was previously used in semi-rigid seat tests with PMHS (Uriot et al. [12], Trosseille et al. [17], Richardson et al. [15]) and can therefore be meaningful for comparisons. Its deltaV is 50km/h and, initially, Uriot et al. [12] derived this pulse from a 56km/h full-width USNCAP test by scaling it down to reduce the risk of injury in the PMHS. The Pulse #2 was selected to represent more strenuous loading conditions. It corresponds to a 56 km/h EuroNCAP MPDB/XT-ADAC test for a compact car.

The environment model will be released under an open-source license to facilitate comparisons by other researchers.

### Model verifications

**Fig 3:**
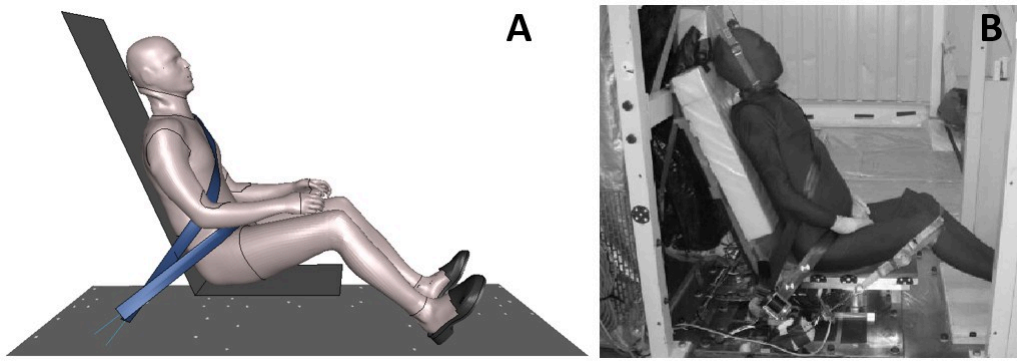
Luet et al. test setup. (A) Numerical test setup. (B) Luet et al. experimental setup [18].

The performance of the environment and occupant models were assessed in both standard and reclined configurations. First, as reclined configurations could lead to occupant submarining, the performance of the GHBMC M50-O model was assessed in an upright submarining configuration on a flat rigid seat from Luet et al. [18] (Fig 3). The occupant was seated on a horizontal seat pan and subjected to a 40 km/h pulse. The occupant was restrained using a four-point seat belt without pretensioners or load limiters. The feet were constrained using rigid overshoes attached to the footrest. The model height was first isotropically scaled by a factor of 0.95 to match the average PMHS stature, and the flesh material density was decreased to reach the average PMHS mass.

**Fig 4:**
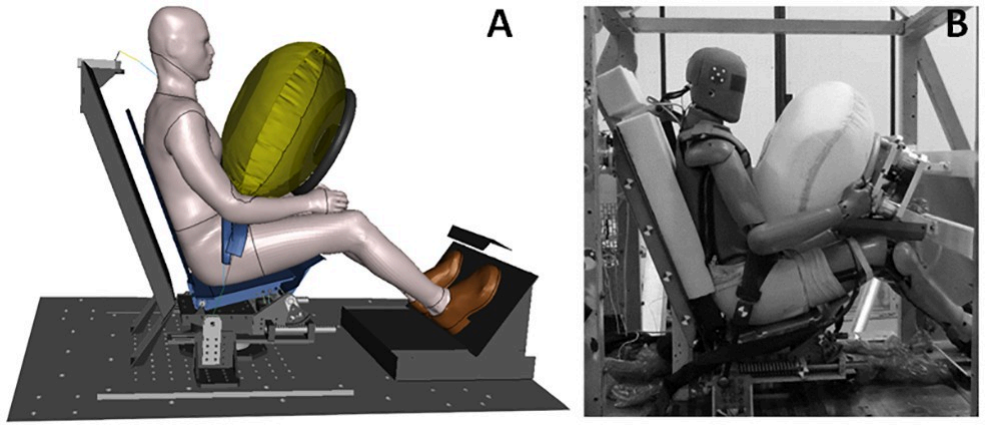
Trosseille et al. test setup. (A) Numerical test setup. (B) Trosseille et al. experimental setup [17].

Then, the semi-rigid seat environment was assessed against the non-submarining Trosseille et al. [17] PMHS test data (Fig 4). The GHBMC M50-O model was scaled to the average height of the PMHS used in Trosseille et al. [17] (factor 0.94) and subjected to the same loading (Pulse #1, Fig 2). The occupant was restrained using a three-point belt combined with a pre-inflated 54-liter airbag. Shoulder and lap belts were both equipped with pretensioners, with time to fire set to 18 and 25ms, respectively. The shoulder belt had a load limiter set to a 3.5kN peak followed by a plateau at 2kN.

**Fig 5:**
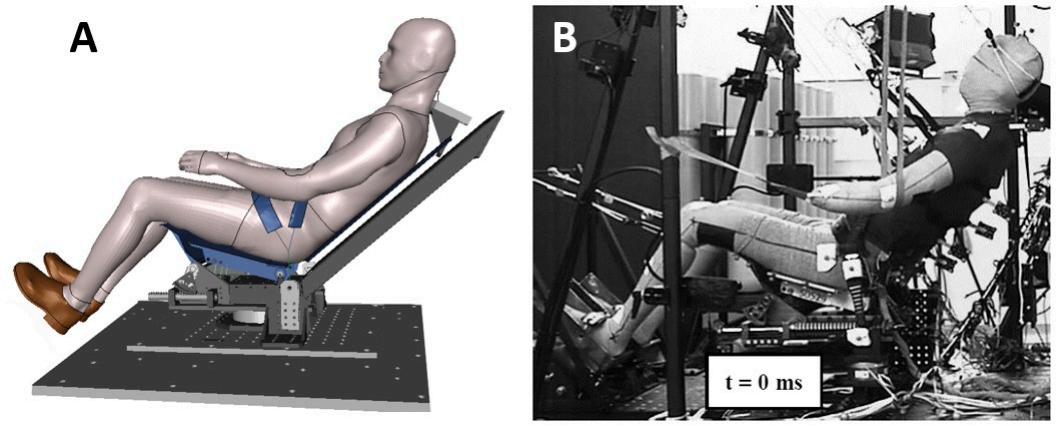
Richardson et al. test setup. (A) Numerical test setup. (B) Richardson et al. experimental setup [15].

Finally, the occupant model was assessed in a reclined configuration using Richardson et al. [13–16] PMHS test data (Fig 5). The environment was composed of the semi-rigid seat with a 15 degrees seat pan and a 50 degrees seatback. The GHBMC M50-O model was repositioned using the PIPER software (v1.0.2) and the metadata provided by the PIPER open-source project for the GHBMC M50-O v5.0. Both are freely available online (www.piper-project.org). Model positioning was performed using the pre-positioning module, with modification of the pelvis and spine positions to match PMHS initial positions (67, 56, 59, 50, 32, 17 degrees for the pelvis, L3, L1, T11, T8, and T1 angles, respectively) [14]. The flesh material density was decreased to reach the average PMHS mass. Finally, the model was subjected to gravity on the reclined semi-rigid seat and then subjected to the same loading as in Richardson et al. [13–16] (Pulse #1, Fig 2). The restraint consisted of a three-point seat belt, with a shoulder load limiter set to 3.5kN. The seat belt was equipped with a dual lap belt pretensioners and a shoulder belt pretensioner. The times to fire were set to 3, 10, and 10ms for the buckle, outboard lap belt, and shoulder belt pretensioners, respectively. Additionally, Richardson et al. [13] provided the position of the lap belt with respect to the pelvis, and the position of the H-point, assessed by palpation of the greater trochanter. In the PMHS tests, the lap belt was initially positioned 89.0 ± 27.9 mm forward and 36.5 ± 16.6 mm upward the ASIS (A_Belt = 67.2 ± 5.4 degrees) and the H-point at 113 ± 12 mm forward and 180 ± 5 mm upward (mean of left and right distances from the edge of the seat). In the numerical model, the lap belt was placed 94mm forward and 44mm upward the ASIS (A_Belt: 66.2°), and the H-point location was approximately at 95mm forward and 160 upward from the edge of the seat (assuming a trochanter landmark at node 7126192 near the center of the trochanter lateral aspect).

The correlation and analysis (CORAplus, v4.0.4) method was used for quantitative evaluation of the model accuracy. However, the number of PMHS was small in most configurations, with sometimes only two PMHS for some signals. This does not allow to estimate reliably a standard deviation for the response as used internally by the CORA corridor method. Therefore, only the CORA correlation score was computed and is provided as a global indicator of the distance between the model responses and the PMHS curves which are available.

### Postures and model repositioning

Ten occupant postures (one baseline and nine reclined) were used. Model repositioning from the baseline was performed using the PIPER software (v1.0.2) pre-positioning module. After that, the model included inverted elements, i.e. with faces intersecting each other, which prevented the simulation from running. Therefore, a smoothing procedure was used to improve the overall mesh quality and remove the inverted elements. First, the mesh was smoothed using the PIPER transformation-smoothing module in the regions that were changed. The smoothing procedure was a combination of surface smoothing and transformation smoothing [19]. Finally, when a few elements were still inverted, additional smoothing was performed in LS-Prepost v4.7 (LSTC, Livermore, CA) and the remaining offending elements were renumbered (i.e. the order of the nodes was modified to remove the face intersection).

The Baseline aims to represent a non-driving occupant sitting on a current vehicle seat configuration. The nine reclined occupant postures correspond to three seat pan angles times three pelvis angles. The seatback angle was fixed to 40 degrees from the vertical for all reclined configurations as it corresponds to the middle of the range used in previous simulation studies (e.g. Gepner et al. [7]) while allowing new activities [20].

Theodorakos et al. [9] conducted a volunteer study to quantify the most comfortable seat pan angle for different backrest angles. For each seatback angle, the seat pan angle was initially set to -5 or +20 degrees from the horizontal, and the participants were instructed to adjust the seat pan inclination until they reached a comfortable positioning. For a 40 degrees seatback, self-selected seat pan angles were from 8.3±7.4 to 20.2±4.6 degrees, for initial angles at -5 and 20 degrees, respectively. In order to cover this range of preferred angles, three seat pan angles (5, 15, and 25 degrees) were selected in the present work, named SP05, SP15, and SP25, respectively. In the absence of actual automotive reclined seats, examples of rotation centers of the seat back and seat pan about the Y-axis are missing. For the current study, the center of rotation was selected arbitrarily on the axis corresponding to the intersection of the seatback and seat pan planes. This point was positioned at 246 mm from the ground.

The upper shoulder belt anchorage point (D-ring side) was attached to the seatback and rotated with it to follow the thorax. Both lap belt anchorage points were rotated with the seat pan.

In order to follow the occupant’s head and to limit the thigh contact with the steering wheel for high seat pan angles, the airbag position was defined relative to the occupant H point for the reclined configurations. The airbag position was set to avoid contact with the occupant’s thigh in the SP25 configuration, then the distance between the hip centers and the airbag center was kept constant in all reclined configurations (i.e. Δx = 195mm; Δz = -485mm). For each reclined position, the occupant knee angle was kept around 110 degrees [21]. The footrest position was then adjusted along the X-axis.

**Fig 6:**
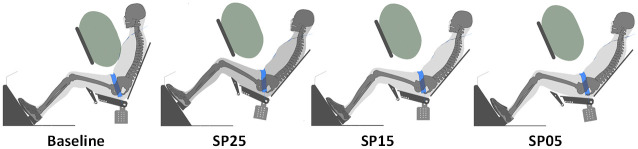
Initial occupant position. For each seat configuration, only the reference occupant posture is represented.

For the HBM repositioning, the baseline GHBMC model was first rotated with the seatback. Then, the pelvis angle was kept as is (around 70 degrees, called Reference) or adjusted by +10 degrees (around 80 degrees, Slouched) or -10 degrees (around 60 degrees, Upright). This 20 degrees range was selected to match the variability observed by Reed and Ebert [11] for a 15 degrees seat pan. In the absence of experimental reference, the same pelvis variation range was used for the other seat pan angles. The model repositioning procedure is detailed in the S1 Table. All models were subjected to gravity on the reclined semi-rigid seat (Fig 6).

**Fig 7:**
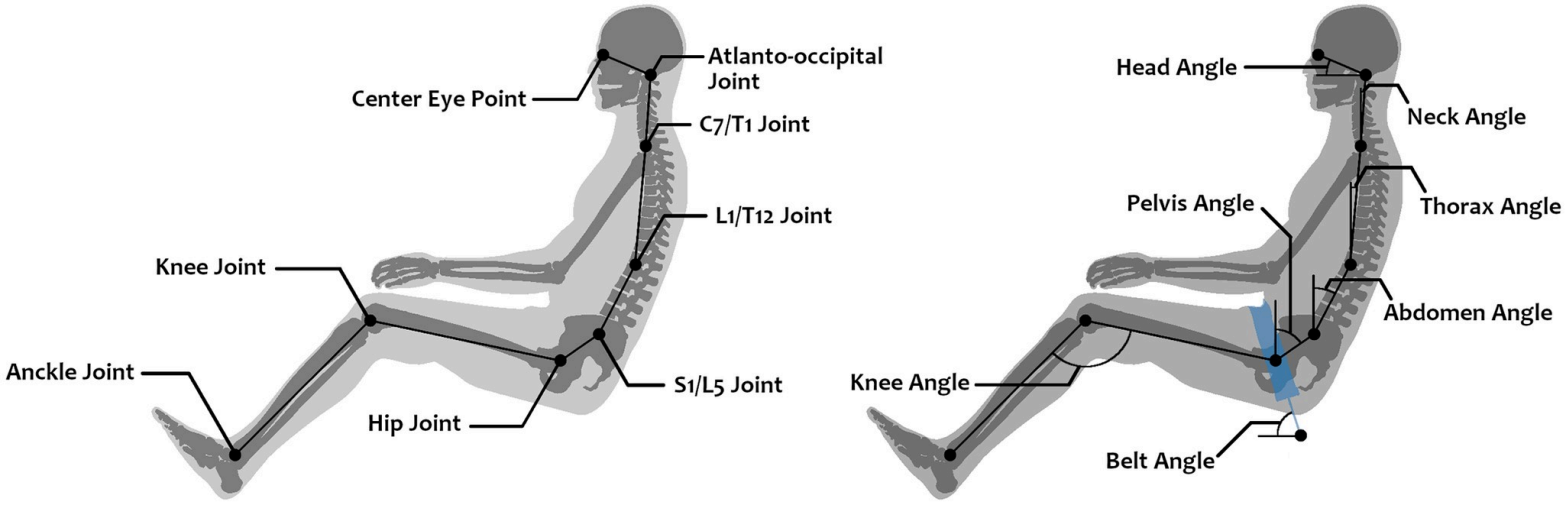
Occupant posture definition. Angles are calculated using the landmarks, and joints defined in the PIPER GHBMC metadata. See Table 1 for values.

To document the posture and facilitate comparisons with future studies, the occupant posture was defined as illustrated in Fig 7. Table 1 summarizes the main angular positions for each posture after the belt slack removal phase (t = 50ms). For the injury analysis, criteria were measured using the instrumentation provided with the model GHBMC manual [22].

**Table 1:** Angular positions (in degrees) for the ten selected postures after the belt slack removal phase (t = 50ms).

	Angle (in degree)						
	Head	Neck	Thorax	Abdomen	Pelvis	Knee	Belt
**Baseline**	20.6	4.4	3.8	27.7	52.1	123.8	75.3
**SP25 Upright**	39.3	23.3	22.2	46.5	59.5	111.7	89.8
**SP25 Reference**	38.2	22.6	23.1	46.0	69.1	112.0	91.5
**SP25 Slouched**	39.8	23.8	22.6	48.7	77.5	109.5	91.7
**SP15 Upright**	38.6	22.7	22.8	45.8	59.9	116.6	85.3
**SP15 Reference**	38.8	23.2	23.0	46.9	69.9	116.8	84.9
**SP15 Slouched**	38.9	23.2	22.9	48.7	79.0	115.3	85.9
**SP05 Upright**	39.1	23.1	23.0	46.9	58.0	116.1	82.7
**SP05 Reference**	38.5	22.6	22.8	46.4	67.8	117.1	81.2
**SP05 Slouched**	39.0	23.3	22.5	50.2	76.2	112.3	79.2

See Fig 7 for angle definitions.

### Restraint variations

Three restraint variations were tested. It was first assumed that the airbag would remain in case of reclined occupants but as several ongoing studies along with Richardson et al. [13–16] do not include airbags, the simulations were repeated without it (Pulse #1) to assess whether the airbag could affect the submarining risk. Second, the modification of the seat pan (i.e. 5 and 25 degrees) was initially performed assuming that the lap belt anchorages would rotate with the seat pan. In the absence of actual automotive reclined seats, the future position of the lap belt anchorages is unknown. As Boyle et al. [5] highlighted that the lap belt angle affected submarining, the simulations with modification of the seat pan (i.e. 5 and 25 degrees) were performed without rotating the lap belt anchorage points (Pulse #1) to dissociate the effect of the seat pan angle and the belt anchorage position on submarining. Other positions could be tested in the future. Finally, reclined configurations with modification of the seat pan angle were performed with both restraint variations (Pulse #1).

## Results

### Model verifications

**Fig 8:**
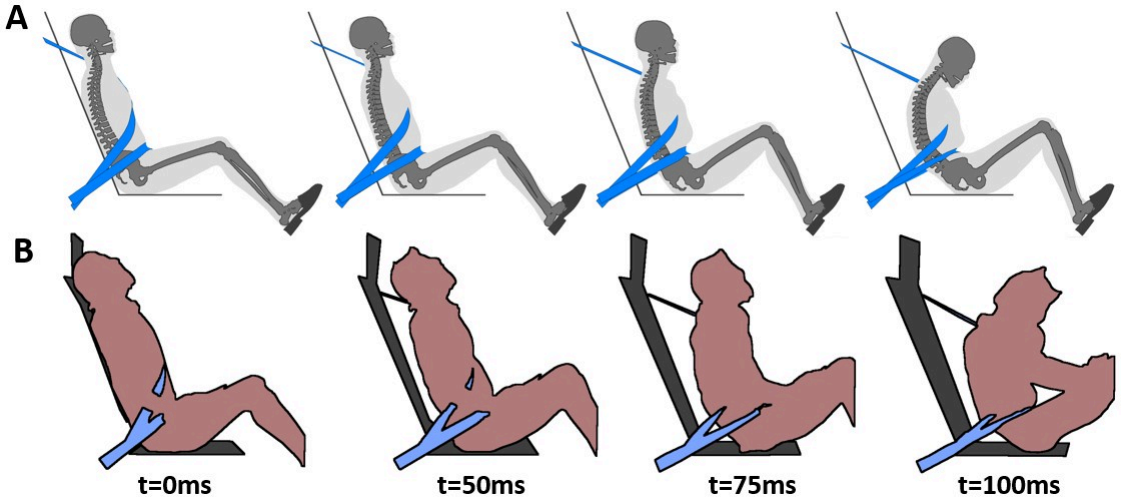
Occupant kinematics in Luet et al. test configuration. (A) Occupant model kinematics. (B) PMHS kinematics (#631) from Luet et al. [18].

Fig 8 illustrates the kinematics of both the numerical model and a PMHS in the Luet et al. test setup [18]. Under these conditions (Config 1, Luet et al.), submarining occurred in all PMHS tests (n = 3), between 80 and 100ms. Similarly, the occupant model submarined around 80ms (Fig 8). Anterior-Superior Iliac Spine (ASIS) fractures occurred for 2 subjects out of 3 but were not observed in the model. Model kinematics and restraint responses seemed to capture the experimental trends (S1 Fig, CORA correlation around or above 0.8).

**Fig 9:**
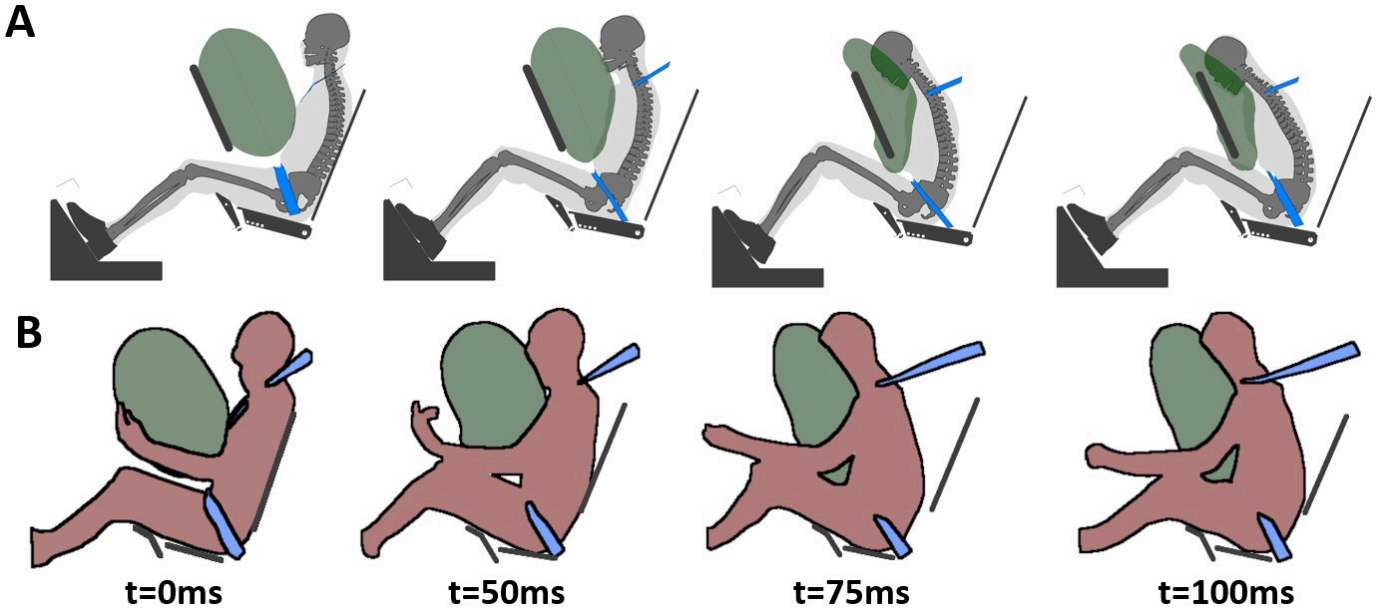
Occupant kinematics in Trosseille et al. test configuration. (A) Occupant model kinematics. (B) PMHS kinematics (non-submarining test, #TOL_THO_07) from Trosseille et al. [17].

Fig 9 illustrates the kinematics of both the numerical model and a PMHS for the Trosseille et al. [17] setup. Under these conditions, the occupant model did not submarine, neither did the PMHS (n = 3). Anterior-Superior Iliac Spine (ASIS) fractures occurred for 2 PMHS out of 3 and were also observed in the model. Both seat and human responses (S2 Fig) captured the experimental trends (CORA correlation around or above 0.8).

**Fig 10:**
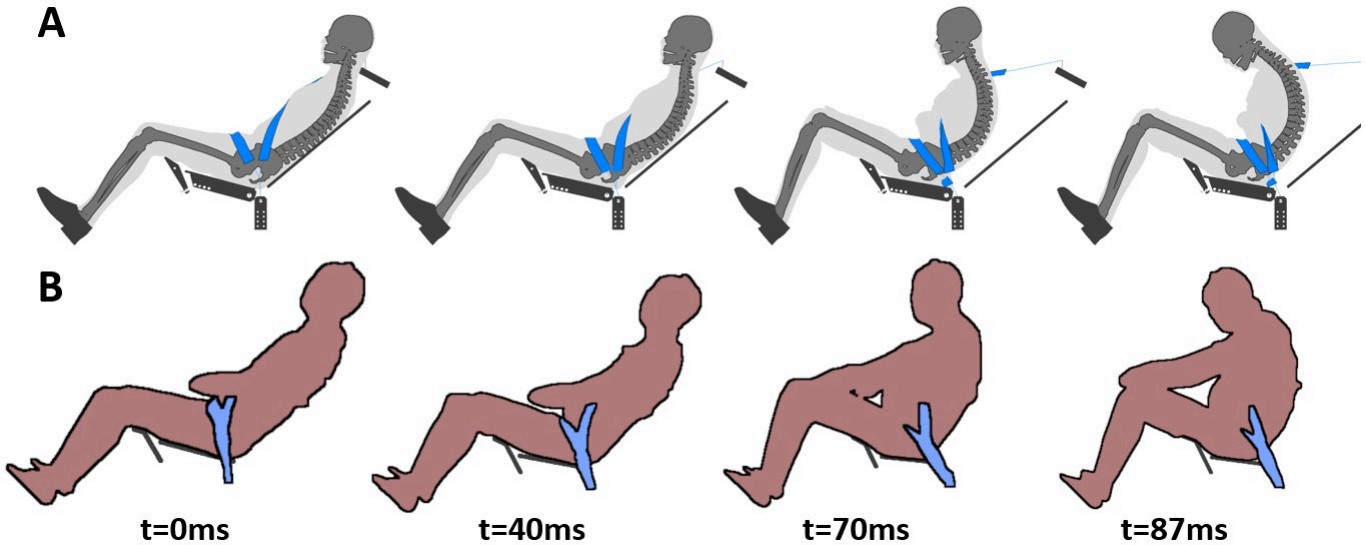
Occupant kinematics in Richardson et al. test configuration. (A) Occupant model kinematics. (B) PMHS kinematics (non-submarining test, #529) from Richardson et al. [16].

Fig 10 illustrates the kinematics of both the numerical model and a PMHS for the Richardson setup [13]. Under these conditions, submarining did not occur for 4 out of 5 PMHS and was not observed for the model. Concerning bone fractures, multiple rib fractures were identified in the PMHS tests (from 0 to 22), as well as sternum fractures (all PMHS), lumbar spine fractures at L1 (3 of 5 PMHS), pelvis fractures at the ASIS level (2 of 5 PMHS), and sacral fractures (4 of 5 PMHS). The model sustained sternum fractures, as well as pelvis fractures (ASIS and sacrum). Overall, the model kinematics and restraint responses described the experimental trends (S3 Fig, CORA correlation above 0.75).

When considering together the three verification setups, it can be observed that the model and environment are able to capture the main trends observed in the experimental tests with correlation scores around 0.8.

### Reclined seating simulations

About half of the forty-one simulations performed terminated before the 200ms simulation time due to negative volume errors close to the pelvis (Table 2). All were reclined, and most cases resulted in submarining. The termination time was over 130 ms in all cases but one (129ms, SP05 Slouched Pulse #2). This was sufficient to reach the maximal occupant forward excursion, and hence to assess the submarining status as submarining would be unlikely in the rebound phase. For simulations that terminated without error, the peak for all injury criteria (e.g. lumbar forces, thorax compression) was observed before the occupant reached the maximal forward excursion. As all the simulations performed presented similar loading mechanisms, the maximal forward excursion time was assumed sufficient to reach the peak for most injury criteria. Some SP05 simulations terminated before reaching the maximal forward excursion, which leads to underestimated injury risks, especially for the head and neck criteria. These simulations were therefore not included in the upper body analysis. The stability of the repositioned occupant models should be further investigated, especially for large occupant excursion (e.g. SP05 positions).

**Table 2:** Termination times (out of 200 msec.) for all studied cases.

Environment	Pulse	SB_A (degrees)	SP_A (degrees)	Occupant Posture				Completion rate (Config.)
				Baseline	Upright	Reference	Slouched	
Baseline ^A^	Pulse #1	22	15	200	-	-	-	100%
		40	25	-	188	200	200	98%
			15	-	150	200	200	92%
			5	-	200	146	148	82%
	Pulse #2	22	15	200	-	-	-	100%
		40	25	-	197	200	150	91%
			15	-	200	189	186	96%
			5	-	146	140	129	69%
Without Airbag ^B^	Pulse #1	40	25	-	168	190	131	82%
			15	-	200	200	200	100%
			5	-	197	136	146	80%
Lap anchor. fixed ^C^	Pulse #1	40	25	-	187	200	200	98%
			5	-	145	150	143	73%
Without Airbag & lap anchor. fixed ^D^	Pulse #1	40	25	-	190	200	148	90%
			5	-	197	150	145	82%
Completion rate (Occupant posture)				100%	91%	89%	82%	-

SB_A: Seatback angle; SP_A: Seat pan angle.

^A^ Configuration included an airbag, and the lap belt anchorage points were rotated with the seat pan if needed;

^B^ Configuration did not include an airbag, and the lap belt anchorage points were rotated with the seat pan if needed;

^C^ Configuration included an airbag, and the lap belt anchorage points were not rotated with the seat pan.

^D^ Configuration did not include an airbag, and the lap belt anchorage points were not rotated with the seat pan.

#### Occupant kinematics and submarining

**Fig 11:**
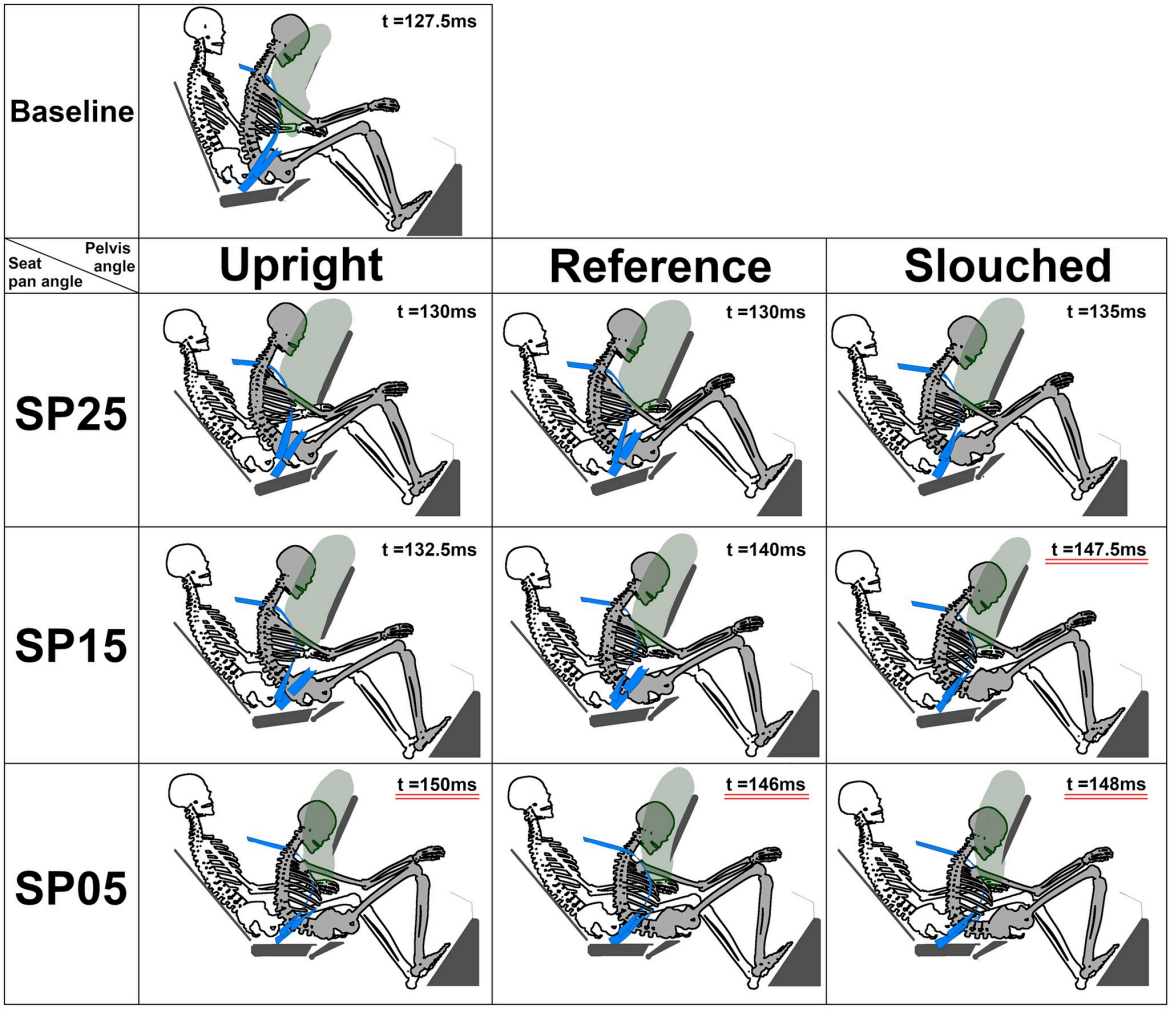
Skeletal position at maximum pelvis excursion under Pulse #1. For each configuration, the occupant skeleton (initial skeleton posture represented in white, while the final posture is represented in grey), the airbag, the seatbelt, and the environment (i.e. seat, footrest, and steering wheel) are represented. The time is underlined when submarining occurred.

The results obtained with the baseline environment (i.e. including an airbag, and with a rotation of the lap belt anchorage points when the seat pan angle was modified) will first be described. For Pulse #1, Fig 11 illustrates the skeleton position when the occupant pelvis reached the maximum forward displacement. Submarining occurred in four of the nine reclined cases and was associated with low seat pan or high pelvis angles. SP15 was close to the submarining limit, as submarining did not occur in SP15 Reference but occurred in SP15 Slouched. SP05 induced submarining in all cases. Increasing the initial pelvis angle led to a higher lap belt penetration into the abdomen (77, 85, and 91mm for the SP05 Upright, Reference, and Slouched, respectively).

Baseline forward excursions were smaller than the reclined ones (S2 Table). For all reclined configurations, the maximum pelvis forward displacement showed a higher variability (216-385mm) than the head and T1 forward displacements (597–660 and 406–436, respectively). These variations for the pelvis displacement were mainly attributed to the seat pan angles (216–244, 265–298, and 368-385mm for SP25, SP15, and SP05, respectively). The seat pan angle also had some effect on the head and T1 upward displacements, reflecting differences in the head contact with the airbag (e.g. for SP25 the head contacts the upper part of the airbag while for SP05, the head contacts the lower part). Additionally, for a given seat pan angle, pelvis forward excursion increased with increasing pelvis initial angles (e.g. for SP25, 216, 218, and 244mm for the Upright, Reference, and Slouched position, respectively).

Compared to Pulse #1, Pulse #2 increased the number of submarining cases (Table 3). Submarining did not occur for the Baseline but it occurred at least once for each seat pan angle in reclined configurations. The effect of the seat pan and the pelvis angle was also visible as in Pulse #1. Decreasing seat pan and increasing pelvis angles decreased the time of submarining for both pulses, exposing the abdomen to the loading of the lap belt for longer durations. Pulse #2 increased the occupant forward excursion for all cases, as well as the head and T1 upward excursions for the reclined position, leading to a contact between the occupant’s head and the steering wheel for the SP25 Upright.

**Table 3:** Comparison of submarining status and occurrence time (in ms) between the two pulses.

	Pulse #1			Pulse #2		
	Upright	Reference	Slouched	Upright	Reference	Slouched
**SP25**	-	-	-	-	-	115–120
**SP15**	-	-	120–125	-	120–125	107.5–112.5
**SP05**	120–125	115–120	110–115	60–65	107.5–112.5	97.5–102.5

#### Injury values

For the Pulse #1, all HIC15 values were below 450 but they varied from 208 for the Baseline to 214–322, 245–371, and 368–411 for the SP25, SP15, and SP05, respectively (S3 Table). Lower seat pan angle seemed associated with higher HIC15. After removing SP25 Upright where contact with the rigid steering wheel led to a HIC15 value of 1355, similar trends can be observed with Pulse #2 as detailed in the S3 Table. The effect of the configuration seemed more limited on the BrIC (variation from 0.47 to 0.59 for the Pulse #1) and the Nij (variation from 0.34 to 0.45 for the Pulse #1).

**Fig 12:**
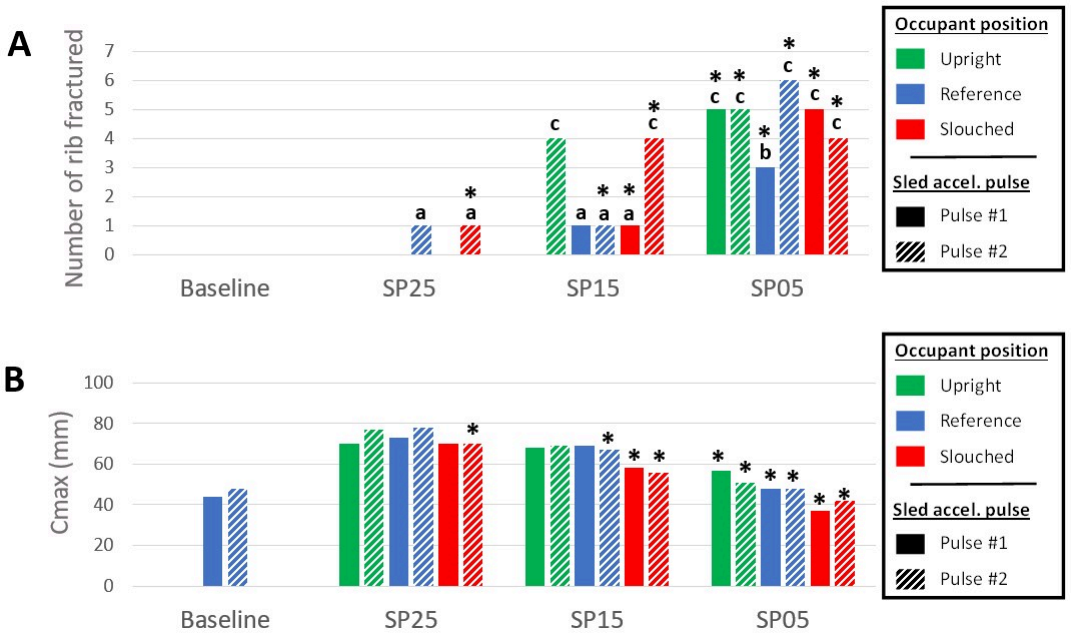
Thorax injury criteria. (A) Number of ribs fractured (a: AIS1; b: AIS2; c: AIS3). (B) Maximum peak deflection Cmax. The green, blue and red bars represent the upright, reference, and slouched occupant initial position, respectively. The filled bars illustrated the results for the Pulse #1, and the hatched bars the results for the Pulse #2. Configurations where submarining occurred are designated with an asterisk.

**Fig 13:**
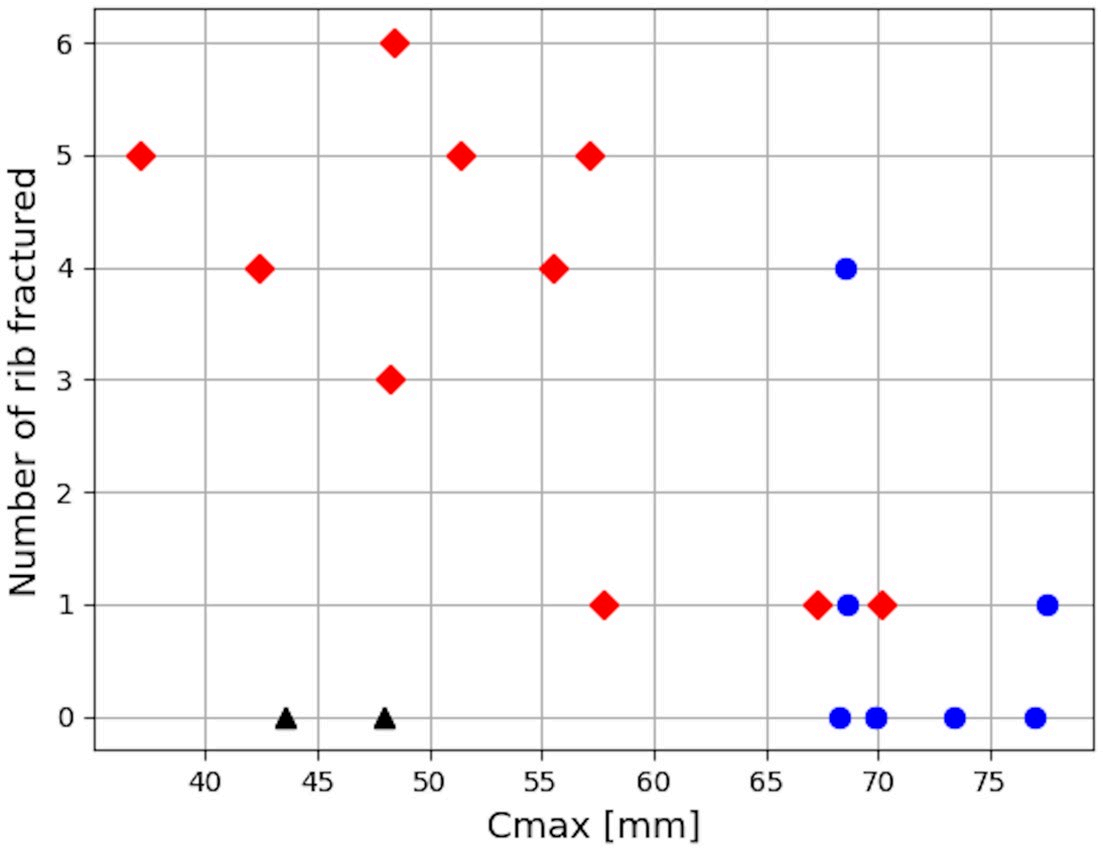
Number of ribs fractured versus the maximum peak deflexion Cmax. Both pulses are represented. The black triangles represent the baseline conditions, while the blue circles and the red diamonds represent reclined positions without and with submarining, respectively.

Reclined postures induced more rib fractures than the Baseline (0–5 vs none for Pulse #1), as illustrated in Fig 12A. Submarining was associated with a higher number of rib fractures, with ranges of variations of 0–1 when submarining did not occur, and 1–5 when it did. Pulse #2 increased the number of ribs fractured for reclined positions, whether submarining occurred or not. Based on their location (i.e. mostly on the right side, from the 5^th^ to the 9^th^ rib), and fracture time, rib fractures seem to be associated with the diagonal belt. In order to see if a global chest deflection could describe the trends in terms of fractured ribs, the occupant chest deflection was initially computed using two measurement points, as described in the GHBMC manual [22]. However, this criterion does not seem to capture properly the compression of the lower chest, especially when submarining occurs. Therefore, the Maximum Peak Deflection computed based on four measurement points (Cmax) was used [23], as it seemed more likely to be sensitive to the various loading locations. The points were positioned in a similar location as in the THOR dummy. The Maximum Peak Deflection was overall higher for reclined cases but there was no clear relationship between Cmax and the number of fractures (Figs 12 and 13). Submarining cases with Cmax below 58 had three or more rib fractures while reclined non-submarining cases had Cmax above 68 and one rib fracture at most in all cases but one (Fig 13). The PC score (S3 Table and S4 Fig) did not show a better trend against the number of fractures.

**Fig 14:**
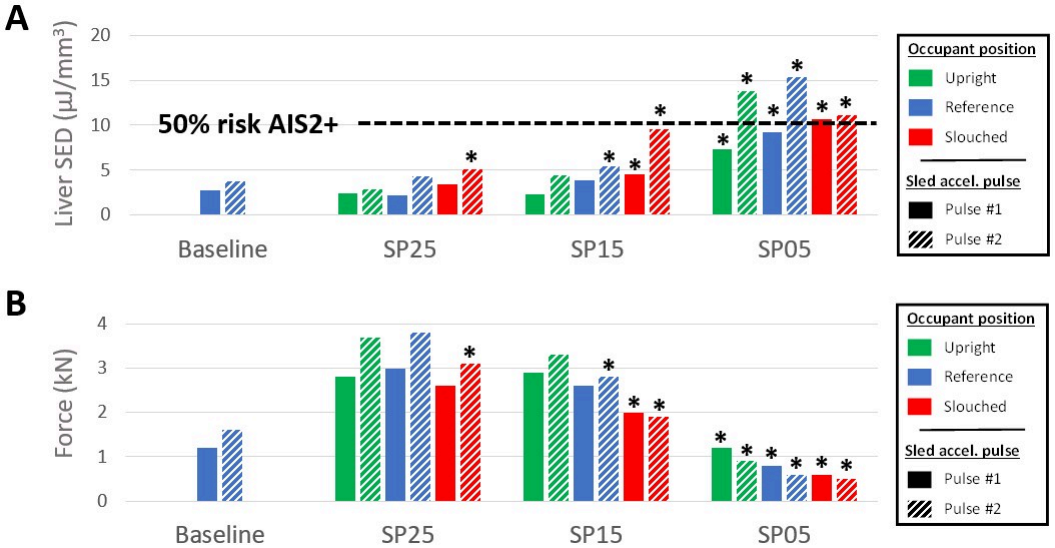
Lumbar and abdominal injury criteria. (A) Liver strain energy density. (B) Normal lumbar forces. The green, blue and red bars represent the upright, reference, and slouched occupant initial position, respectively. The filled bars illustrated the results for the Pulse #1, and the hatched bars the results for the Pulse #2. Configurations where submarining occurred are designated with an asterisk.

The seat pan angle, occupant posture, pulse, and submarining status all affected the liver strain energy density (SED) (i.e. strain energy computed in the liver normalized by the initial organ volume [24]) as illustrated in Fig 14A. Additionally, earlier submarining led to higher liver SED. For SP25, liver SED was relatively close to the Baseline (i.e. variations of 2.5 μJ/mm^3^ at most, all values below 6 μJ/mm^3^), although submarining occurred for SP25 Slouched with Pulse #2. For the SP15 seat configuration, liver SED seems particularly sensitive to the occupant pelvis angle (e.g. the SED increased by 4.2 μJ/mm^3^ between SP15 Reference and SP15 Slouched for the Pulse #2). SP05 led to the highest liver SED (greater than 10 μJ/mm^3^ for the Pulse #2). For the Pulse #2, this criterion indicated a risk of AIS2+ liver injury of 2.0, 3.5, 16, and 62%, for the Baseline, SP25, SP15, and SP05 configurations respectively [24].

Compared to the Baseline (1.3kN for the Pulse #1), lumbar forces decreased when submarining occurred (range of variations of 0.6–2.0kN) but increased otherwise (range of variations of 2.6–3.0kN). The force was also increased when increasing the seat pan angle or diminishing the pelvis angle (Fig 14B).

Some small pelvic fractures (i.e. that did not propagate throughout the bone) were observed at both Anterior-Superior Iliac Spines (ASIS) and the coccyx for all occupant postures and both pulses, including the Baseline. The seat pan angle and the occupant posture had a minimal impact on the severity of these fractures (i.e. the volume of eroded elements).

The 3kN shoulder belt load limit on the D-ring side was reached for all simulations. On the buckle side, reclined configurations showed lower shoulder belt forces than the Baseline, especially when submarining occurred (2.7–4.4, and 4.6 kN respectively, for the Pulse #1). Lap belt forces were similar to the Baseline for positions where submarining did not occur, and diminished when it did (S3 Table).

### Restraint variations

For simulations with and without airbag, submarining occurred for the same angles and within 125ms (Table 3). Furthermore, for each reclined configuration removing the airbag had a small impact on pelvis kinematics, with pelvis forward excursion of 219–246, 269–301, and 352-385mm for SP25, SP15, and SP05, respectively (S4 Table). The absence of an airbag allowed for more head excursion and led to a head-to-thigh contact for all seat pan angles. Lumbar forces were the same for both airbag conditions, within 0.6–2.0kN when submarining occurred and within 2.6–3.0kN otherwise.

As the lap belt anchorages were close to the seat pan center of rotation (intersection of the seatback and seat pan planes), rotating or keeping the anchorage points led to a similar initial lap belt position and the cable attachment position only changed by 12 mm. Reclined configurations without rotating the lap belt anchorage points has a limited effect on the response compared to the baseline environment (S4 Table) for both airbag conditions. For the environment with a nominal anchorage location and an airbag, pelvis forward displacements were within 216–243, and 370-385mm for the SP25 and SP15 configurations, respectively. The corresponding displacements were 212–243, and 374-385mm, for the environment with the nominal anchorage position but without an airbag.

## Discussion

Forty-one simulations were performed to analyze the model response in a reclined position by varying seat pan and pelvis angles under two pulses. As the seat pan and pelvis angles were fixed in previous studies, the results can be considered as complementary.

Simulation results suggested that lowering the seat pan angle, increasing the pelvis angle, and increasing the pulse severity all increased the occupant submarining occurrence and the corresponding injury risk. Small variations of these parameters can strongly affect the submarining status and injury risk. For example, for the Pulse #2 at SP15, the Reference position did not lead to submarining and had a small associated risk of injuries, whereas the Slouched posture led to submarining and a 45% risk of AIS2+ liver injury. The most critical condition (Pulse #2, SP05 Reference) led to submarining, an AIS2+ liver injury risk larger than 75%, and six rib fractures. Overall, considering the effect of slouching on the model response, studying experimentally the pelvic position in reclined configuration seems required. This could be done in conjunction with a study of preferred seat pan angles, as the seat pan angle would likely impact the pelvis position. The pelvic and seat pan angles used in the current study were selected based on the combination of a reclined comfort study for airline seats [9] and pelvic angle observations with a fixed seat pan angle (15 degrees) [11].

Higher lumbar normal forces were observed for reclined configurations where submarining did not occur. This is consistent with other numerical studies (e.g. [5]), and the first PMHS data in reclined conditions (L1 fracture observed on 3 PMHS out of 5 in Richardson et al. [14]). These forces increased by 2.1kN between the Baseline and the SP25 Upright positions (Pulse #2). While a lumbar fracture criterion is currently under development for the GHBMC M50-O, it can be observed that all lumbar forces were lower than the fracture forces observed by Arun et al. [25] in pure compression tests of lumbar functional units. However, the contributions of bending moments and the possible relevance for older and female occupants should be further investigated.

Overall, SP15, which corresponds to the seat pan angle used in previous studies (e.g. Gepner et al. [7]), seems to correspond to the transition between proper restraint and submarining associated with significant injury risk (Fig 14A). This suggests that for a 15 degrees seat pan (which is common for upright seating); other parameters may be determinant (e.g. pulse, pelvic angle). This consistent with the kinematic results of Boyle et al. [5] finding an effect of the seat pan design, lower extremity constraints, and restraint parameters (pretensionner and locking tongue, no airbag used) on the response and submarining of the GHBMC M50-OS model. This high sensitivity may also be consistent with the fact that other studies using 15 degrees observed that parameters such as the model size or model type could affect the response [6, 7], and in general, with the difference of results between studies. It seems therefore important to provide the details of the seat design, restraint conditions, and HBM posture and positioning approach to allow comparisons at that angle.

However, the semi-rigid seat used in the current study, which will be used in experimental studies with PMHS was not initially designed for reclined positions. As future HAV interior designs are currently unknown, assumptions had to be made regarding the airbag and anchor belt positions for example. These are likely to evolve in the future. Besides, the simulations performed in the current study suggested that removing the airbag had a minimal impact on the submarining pattern. Both airbag conditions showed similar thorax and lumbar injury risks. As the lap belt anchorage points are close to the seat pan center of rotation in the current setup, the two anchorage locations (rotating with the seat pan or the nominal position) showed similar results. Additional simulations with higher variations of the lap belt angle could be performed to better dissociate the interaction of the seat pan angle and the lap belt location on the occupant restraint.

This study, as the previous ones, is limited by the reclined data available to validate the submarining behavior in reclined configurations. For the current study, the GHBMC response could be assessed in a reclined configuration using the recently published Richardson and al. [13–16] PMHS test data. Although this is an improvement over previous efforts, additional experimental studies including both submarining and non-submarining conditions would be useful to refine the assessment of the model sensitivity to submarining. This refinement may be important considering the sensitivity to submarining and the effect of the seat pan angle observed in this numerical study. Ongoing experimental studies funded by NHTSA in the United States also use the semi-rigid seat but with restraint conditions different from Richardson et al. [13–16] (e.g. no pretensioners and load limiters). This will provide additional results for reclined configurations with a 15 degrees seat pan. PMHS tests in reclined configurations with several seat pan angles are also being prepared (e.g. ENOP project in Europe). Altogether, these data could be used to assess the occupant kinematics in several reclined configurations, and additional validation efforts should be planned as more data becomes available.

For injury, the assessment of the model predictions will also be facilitated by the publication of new experimental data. For now, reclined seating simulations suggested that the abdominal injury risk, estimated using the liver SED, was affected by both the pelvis and seat pan angles. However, Beillas et al. [24] highlighted the importance of both the geometry of the organ in the model and of the loading location to predict injury. As such, this criterion and associated risk curves (which were obtained by simulation) are model specific. Furthermore, the current study highlights that the chest deflection Cmax (that could also be measured on an ATD) was not in line with the number of rib fractures, especially when submarining occurred. This may suggest a different loading mechanism due to the torso angle that is not captured by the criterion. Therefore, the realism of the model rib fracture prediction, as well as the adequacy of ATD criteria should be further investigated to better understand these discrepancies.

## Conclusions

Overall, the simulation results suggest that both kinematics and injuries resulting from reclined configurations with a seatback at 40 degrees are sensitive to the pulse severity, seat pan, and pelvis angles. For a 15 degrees seat pan configuration and a belt restraint using two pretensioners, the pelvis initial angle could lead to either a proper occupant restraint or submarining associated with significant injury risk. It is hoped that some of the parameters found to be sensitive in this study will be investigated in PMHS tests. An experimental campaign of volunteers that could help to define more realistic occupant postures while modifying the seat pan angle in reclined conditions is ongoing at the laboratory. Although the trends observed during the model verification performed for the current study were encouraging (e.g. comparison to the first published reclined data from Richarson et al.), modeling perspectives include additional validation of the model submarining behavior when additional relevant experimental data will be available.

## Supporting information

S1 Fig Model response compared to Luet et al. PMHS test data [18]. (PDF)

S2 Fig Model response compared to Trosseille et al. PMHS test data [17]. (PDF)

S3 Fig Model response compared to Richardson et al. PMHS test data [13–16]. (PDF)

S4 Fig Number of ribs fractured versus PC score (baseline environment). Both pulses are represented. The black triangles represent the baseline conditions, while the blue circles and the red diamonds represent reclined positions without and with submarining, respectively.
(PDF)

S1 Table Human body model repositioning procedure. Model repositioning was performed using the PIPER software (www.piper-project.org). For each occupant posture, the "X" symbol indicates that the given step is done.
(PDF)

S2 Table Body part center of gravity excursions. The forward excursion corresponds to the maximum excursion along the X-axis. The upright excursion corresponds to the one along the Z-axis while reaching the maximum forward excursion, for each body part; a negative value means a downward displacement. The gray rows give the average excursion for each seat configuration. SB_A: Seatback angle; SP_A: Seat pan angle. ^A^ Simulation stopped before reaching the maximum head and T1 forward excursions. ^B^ Simulation stopped before reaching the maximum head, T1, and pelvis forward excursions.
(PDF)

S3 Table Injury criteria for all baseline environment cases. The gray rows give the average for each seat configuration. SB_A: Seatback angle; SP_A: Seat pan angle.
(PDF)

S4 Table Body part center of gravity excursions for restraint variation simulations. The forward excursion corresponds to the maximum excursion along the X-axis. The upright excursion corresponds to the one along the Z-axis while reaching the maximum forward excursion, for each body part; a negative value means a downward displacement. The gray rows give the average excursion for each seat configuration. SB_A: Seatback angle; SP_A: Seat pan angle. ^A^ Simulation stopped before reaching the maximum head and T1 forward excursions. ^B^ Simulation stopped before reaching the maximum head, T1, and pelvis forward excursions.
(PDF)
