# Supplementary material for: Effects of seat pan and pelvis angles on the occupant response in a reclined position during a frontal crash
Source: PLoS One. 2021 Sep 20;16(9):e0257292. doi: 10.1371/journal.pone.0257292 (PMC8452024; doi:10.1371/journal.pone.0257292)
Supplement: S1 Fig — (PDF) [file pone.0257292.s001.pdf]

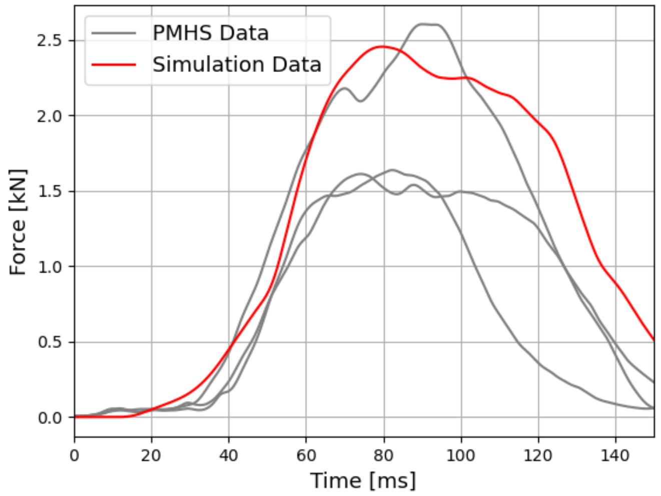

A : Lower shoulder belt forces

Correlation Method Score (CORA) : 0.873

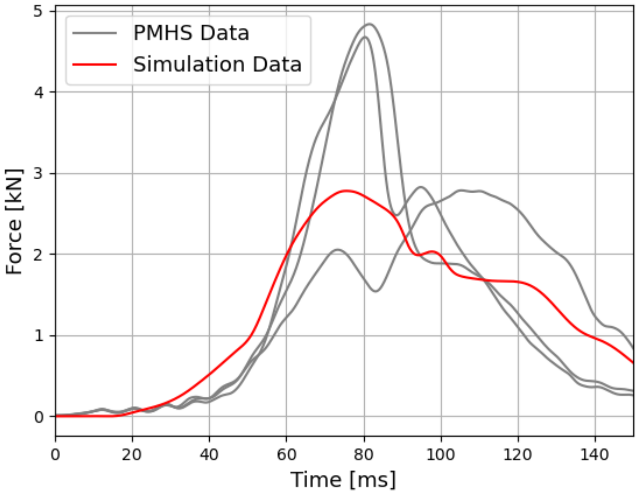

B: Outboard lap belt forces

Correlation Method Score (CORA) : 0.925

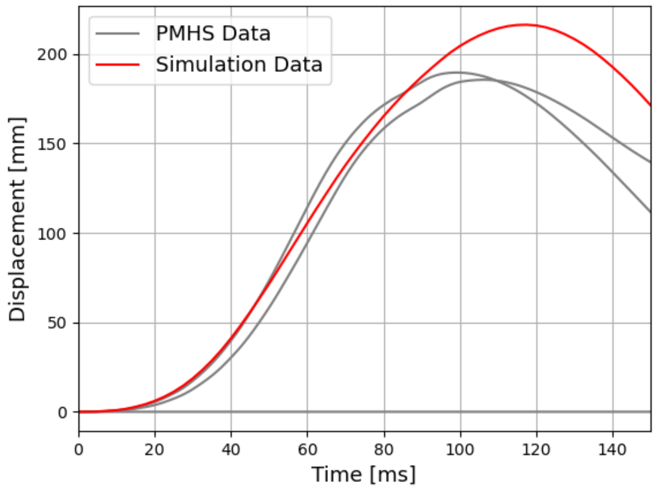

E : H-point X displacement

Correlation Method Score (CORA) : 0.939

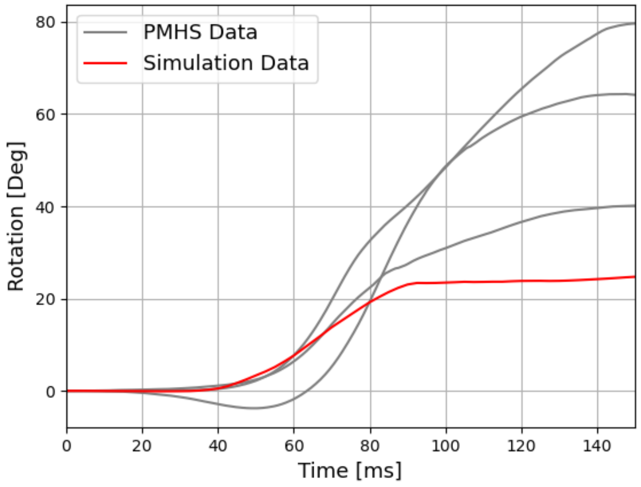

F : Pelvis Y rotation

Correlation Method Score (CORA) : 0.786

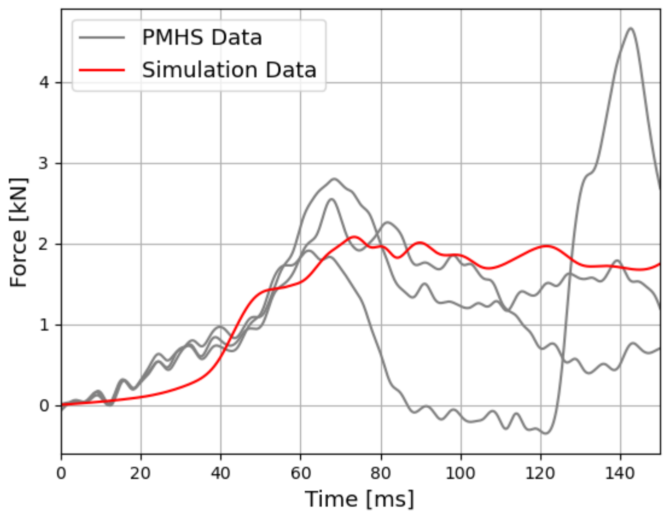

D: Seatpan forces in Z-direction

Correlation Method Score (CORA) : 0.917
