# Supplementary material for: Effects of seat pan and pelvis angles on the occupant response in a reclined position during a frontal crash
Source: PLoS One. 2021 Sep 20;16(9):e0257292. doi: 10.1371/journal.pone.0257292 (PMC8452024; doi:10.1371/journal.pone.0257292)
Supplement: S2 Fig — (PDF) [file pone.0257292.s002.pdf]

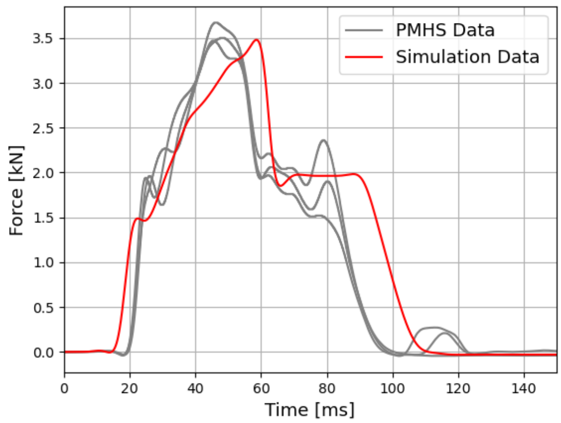

A: Upper shoulder belt forces

Correlation Method Score (CORA) : 0.914

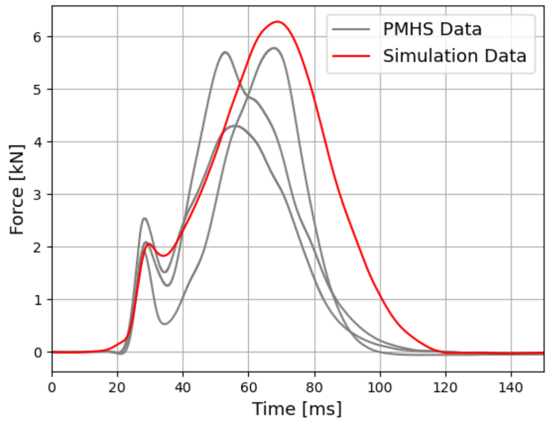

B: Outboard lap belt forces

Correlation Method Score (CORA) : 0.809

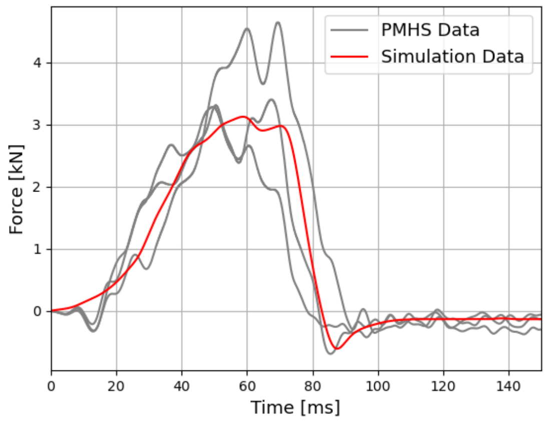

C: Seatpan forces in X-direction

Correlation Method Score (CORA) : 0.982

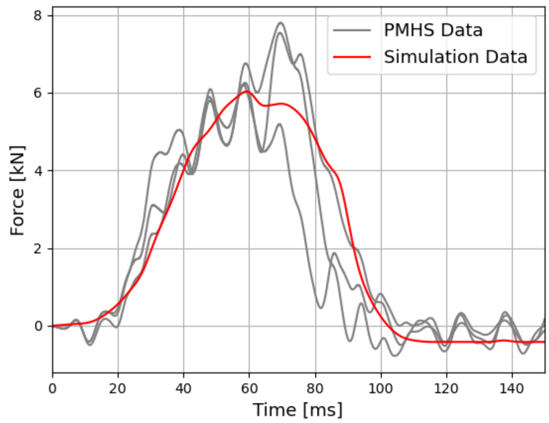

D: Seatpan forces in Z-direction

Correlation Method Score (CORA) : 0.940

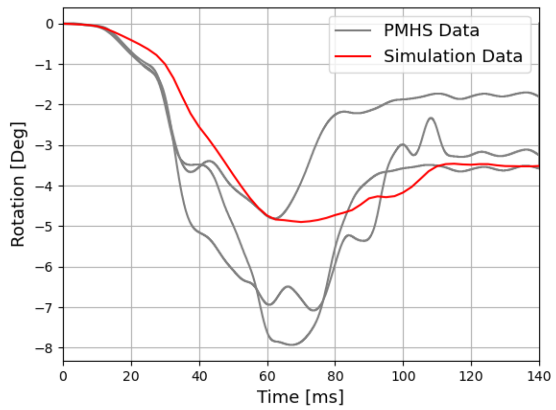

E: Seatpan rotation around the Y-axis

Correlation Method Score (CORA) : 0.839

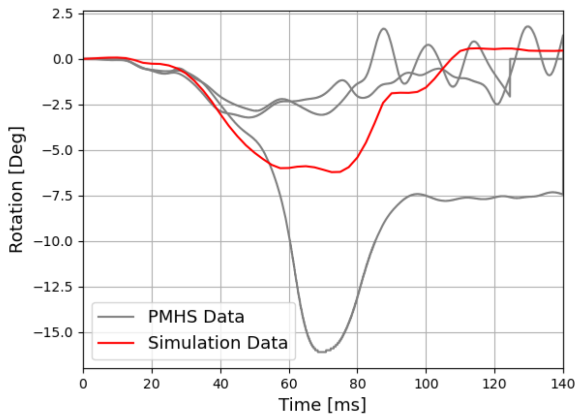

F: Anti submarining guards rotation around the Y-axis

Correlation Method Score (CORA) : 0.896

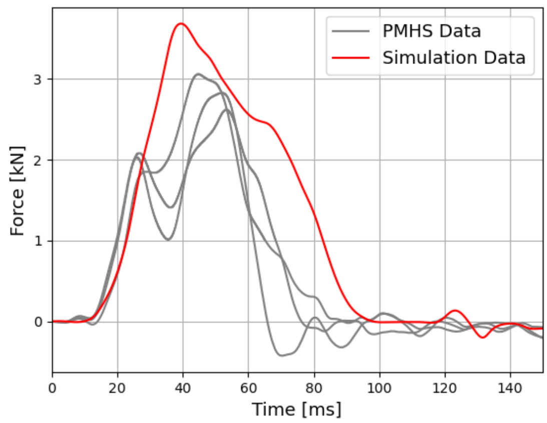

G: Footrest forces in X-direction

Correlation Method Score (CORA) : 0.793

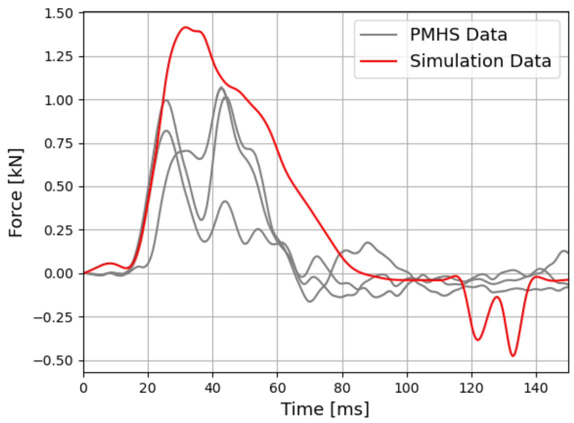

H: Footrest forces in Z-direction

Correlation Method Score (CORA) : 0.782
