# Supplementary material for: Effects of seat pan and pelvis angles on the occupant response in a reclined position during a frontal crash
Source: PLoS One. 2021 Sep 20;16(9):e0257292. doi: 10.1371/journal.pone.0257292 (PMC8452024; doi:10.1371/journal.pone.0257292)
Supplement: S3 Fig — (PDF) [file pone.0257292.s003.pdf]

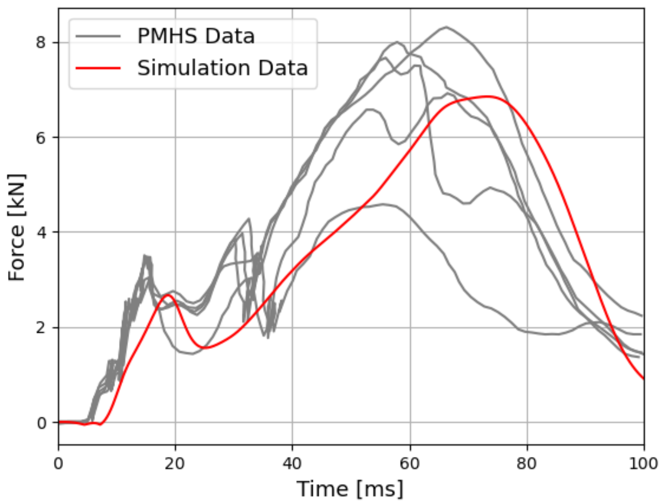

A: Outboard lap belt forces

Correlation Method Score (CORA) : 0.965

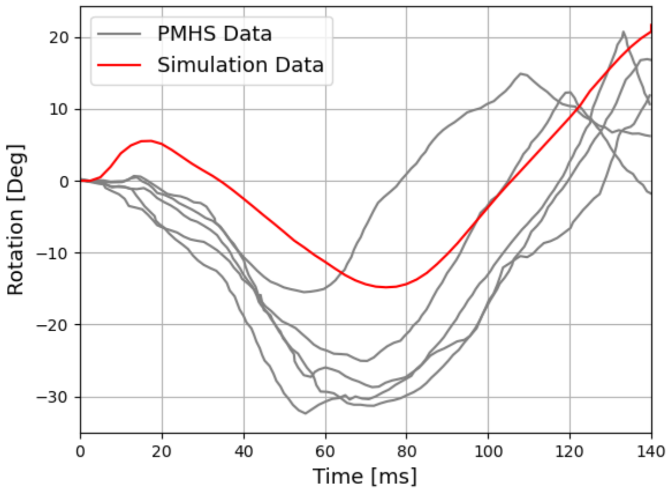

B: Outboard lap-belt angle

Correlation Method Score (CORA) : 0.814

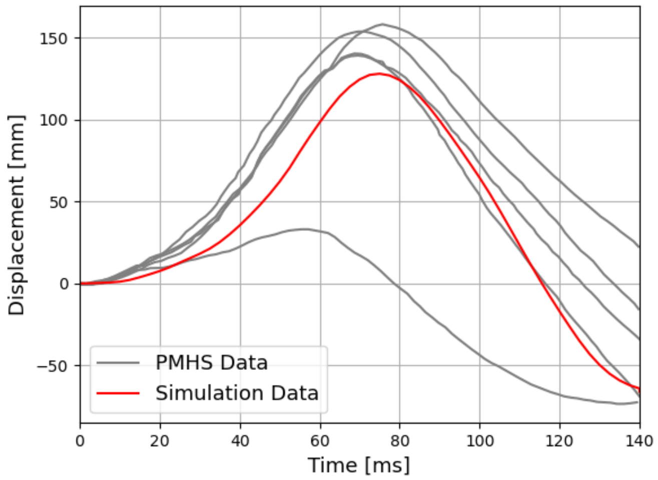

C: Pelvis X displacement

Correlation Method Score (CORA) : 0.981

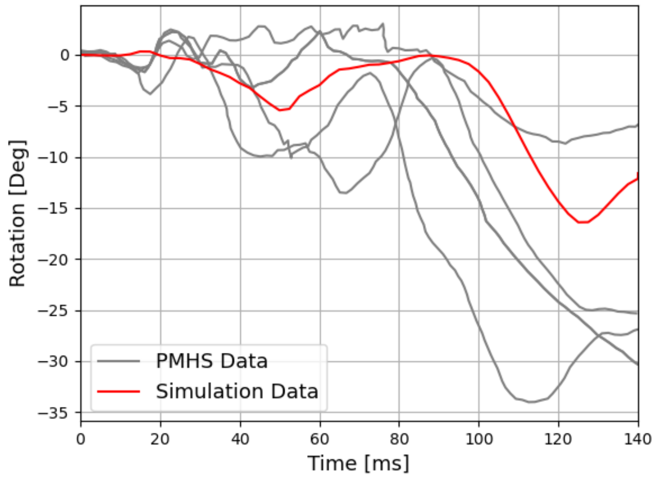

D: Pelvis Y rotation

Correlation Method Score (CORA) : 0.813

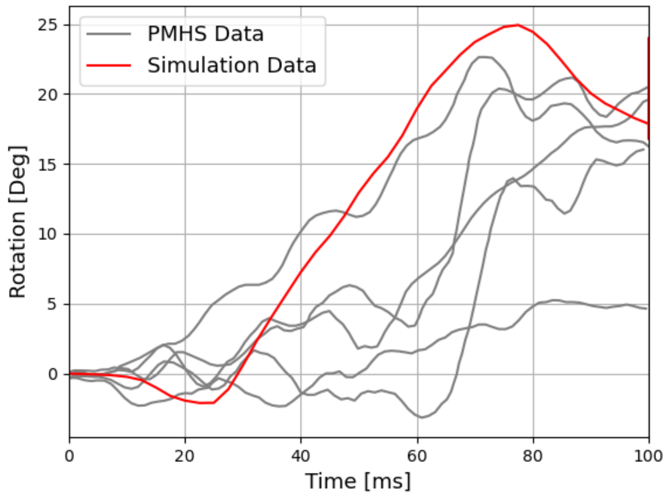

E : T11 Y rotation with respect to L3 Y rotation

Correlation Method Score (CORA) : 0.813

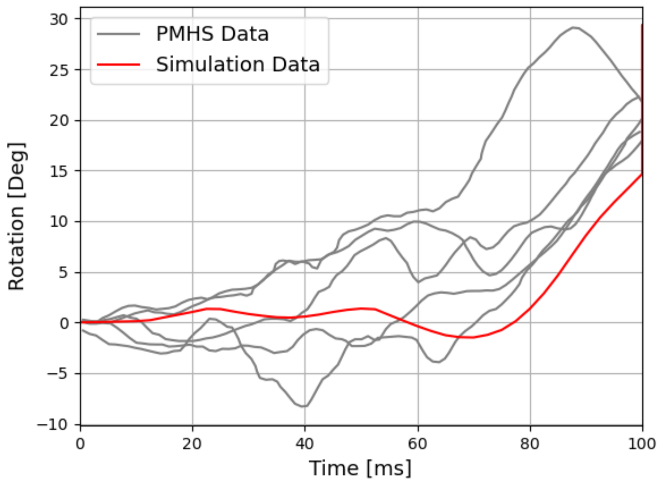

F : Pelvis-L3 Y rotation

(Pelvis-L3 vector relative to the global X-axis)

Correlation Method Score (CORA) : 0.763
