# Supplementary figures and images for: Effects of seat pan and pelvis angles on the occupant response in a reclined position during a frontal crash
Source: PLoS One. 2021 Sep 20;16(9):e0257292. doi: 10.1371/journal.pone.0257292 (PMC8452024; doi:10.1371/journal.pone.0257292)

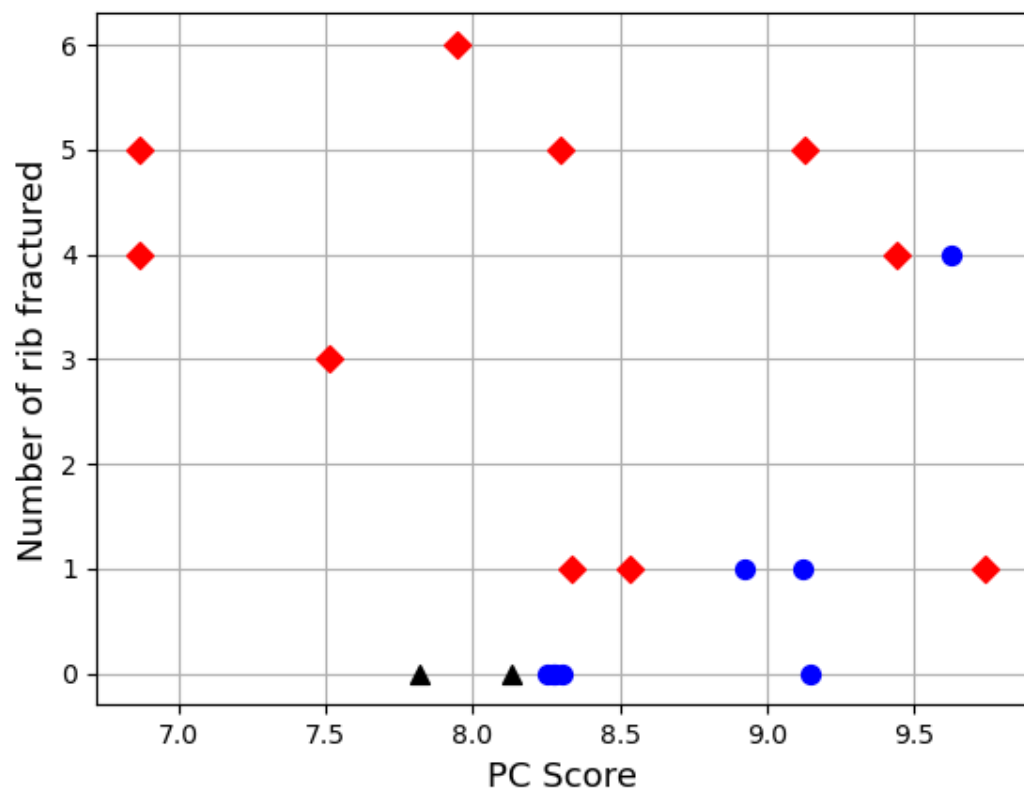

Supplement: S4 Fig — Both pulses are represented. The black triangles represent the baseline conditions, while the blue circles and the red diamonds represent reclined positions without and with submarining, respectively. (PDF) [file pone.0257292.s004.pdf]
