# Supplementary material for: Effects of seat pan and pelvis angles on the occupant response in a reclined position during a frontal crash
Source: PLoS One. 2021 Sep 20;16(9):e0257292. doi: 10.1371/journal.pone.0257292 (PMC8452024; doi:10.1371/journal.pone.0257292)
Supplement: S1 Table — Model repositioning was performed using the PIPER software (www.piper-project.org). For each occupant posture, the "X" symbol indicates that the given step is done. (PDF) [file pone.0257292.s005.pdf]

	Occupant posture			
**Step (in chronological order)**	**Baseline**	**Reclined Upright**	**Reclined Reference**	**Reclined Slouched**
**HBM rotation**<br>**Method**: HBM model was rotated with the seatback<br>**Fixed bones**: /		X	X	X
**Adjust the pelvis angle**<br>**Method**: Changing the bone pelvic angle<br>**Fixed bones**: Head, upper extremities, cervical and thorax vertebrae		X		X
**Adjust the hip angle**<br>**Method**: Adjusting the hip angle until the thighs contacts the seat pan<br>**Fixed bones**: Head, upper extremities, spine, pelvis		X	X	X
**Adjust the knee angle**<br>**Method**: Adjusting the knee angle to 110°<br>**Fixed bones**: Head, upper extremities, spine, pelvis, femurs		X	X	X
**Adjust the ankle angle**<br>**Method**: Adjusting the foot angle until the feet contact the footrest<br>**Fixed bones**: Head, upper extremities, spine, pelvis, femurs, tibia		X	X	X
**Lower the upper extremities (avoid initial penetration with the airbag)**<br>**Method**: Changing the humerus and then the radius angles<br>**Fixed bones**: Head, spine, pelvis, and lower extremities	X	X	X	X
**Model subjected to gravity on the semi-rigid seat**	X	X	X	X
