# Supplementary material for: Effects of seat pan and pelvis angles on the occupant response in a reclined position during a frontal crash
Source: PLoS One. 2021 Sep 20;16(9):e0257292. doi: 10.1371/journal.pone.0257292 (PMC8452024; doi:10.1371/journal.pone.0257292)
Supplement: S2 Table — The forward excursion corresponds to the maximum excursion along the X-axis. The upright excursion corresponds to the one along the Z-axis while reaching the maximum forward excursion, for each body part; a negative value means a downward displacement. The gray rows give the average excursion for each seat configuration. SB_A: Seatback angle; SP_A: Seat pan angle. A Simulation stopped before reaching the maximum head and T1 forward excursions. B Simulation stopped before reaching the maximum head, T1, and pelvis forward excursions. (PDF) [file pone.0257292.s006.pdf]

	Submarining occurrence	Head excursion (mm) Forward	Head excursion (mm) Upright	T1 excursion (mm) Forward	T1 excursion (mm) Upright	Pelvis excursion (mm) Forward	Pelvis excursion (mm) Upright	Lap belt penetration (mm)
**Pulse #1**								
**SB_A=22**	**-**	**444**	**-65**	**332**	**-24**	**202**	**-24**	**-**
**SP_A=15**	**-**	**444**	**-65**	**332**	**-24**	**202**	**-24**	**-**
Baseline	No	444	-65	332	-24	202	-24	-
**SB_A=40**	**-**	**622**	**-74**	**422**	**-14**	**293**	**-16**	**-**
**SP_A=25**	**-**	**639**	**-27**	**417**	**56**	**226**	**5**	**-**
Upright	No	660	-5	436	79	216	3	-
Reference	No	632	-29	409	61	218	5	-
Slouched	No	625	-46	406	28	244	7	-
**SP_A=15**	**-**	**629**	**-62**	**420**	**2**	**279**	**-11**	**-**
Upright [A]	No	646	-45	423	18	265	-21	-
Reference	No	624	-65	418	3	274	-19	-
Slouched	Yes	618	-75	418	-15	298	8	75
**SP_A=5**	**-**	**597**	**-134**	**429**	**-101**	**375**	**-41**	**-**
Upright	Yes	597	-137	431	-107	368	-51	77
Reference [B]	Yes	592	-131	421	-96	373	-41	85
Slouched [B]	Yes	603	-133	434	-99	385	-31	91
**Pulse#2**								
**SB_A=22**	**-**	**443**	**-54**	**345**	**-25**	**247**	**-29**	**-**
**SP_A=15**	**-**	**443**	**-54**	**345**	**-25**	**247**	**-29**	**-**
Baseline	No	443	-54	345	-25	247	-29	-
**SB_A=40**	**-**	**639**	**-52**	**473**	**-26**	**362**	**-14**	**-**
**SP_A=25**	**-**	**667**	**2**	**471**	**52**	**283**	**21**	**-**
Upright	No	682	28	485	78	265	11	-
Reference	No	663	-4	460	56	275	14	-
Slouched	Yes	657	-19	467	22	308	37	78
**SP_A=15**	**-**	**644**	**-48**	**472**	**-17**	**357**	**-5**	**-**
Upright	No	647	-35	472	-2	330	-21	-
Reference	Yes	644	-48	472	-16	346	-7	62
Slouched	Yes	641	-60	471	-34	395	12	89
**SP_A=5**	**-**	**606**	**-109**	**476**	**-113**	**445**	**-57**	**-**
Upright [B]	Yes	615	-117	497	-139	459	-74	92
Reference [B]	Yes	615	-121	486	-119	456	-54	103
Slouched [B]	Yes	587	-89	445	-82	420	-42	90
