# Supplementary material for: Effects of seat pan and pelvis angles on the occupant response in a reclined position during a frontal crash
Source: PLoS One. 2021 Sep 20;16(9):e0257292. doi: 10.1371/journal.pone.0257292 (PMC8452024; doi:10.1371/journal.pone.0257292)
Supplement: S3 Table — The gray rows give the average for each seat configuration. SB_A: Seatback angle; SP_A: Seat pan angle. (PDF) [file pone.0257292.s007.pdf]

	Submarining status	HIC15	BrIC	CSDM	Nij	Cmax (mm)	PC Score	TIC NSF	nb of rib fract.	Liver SED (μJ/mm^3)	Lumbar forces (kN)	Belt forces (kN) Lap Anchor.	Lap Buckle	Shoulder Buckle	Shoulder D-ring
**Pulse #1**															
**SB_A=22**		**208**	**0.50**	**0.23**	**0.39**	**43.6**	**7.82**	**54.2**	**0**	**2.7**	**1.2**	**7.8**	**7.3**	**3.6**	**3.0**
**SP_A=15**		**208**	**0.50**	**0.23**	**0.39**	**43.6**	**7.82**	**54.2**	**0**	**2.7**	**1.2**	**7.8**	**7.3**	**3.6**	**3.0**
Baseline	No	208	0.50	0.23	0.39	43.6	7.82	54.2	0	2.7	1.2	7.8	7.3	3.6	3.0
**SB_A=40**		**323**	**0.52**	**0.03**	**0.40**	**61.1**	**8.23**	**84.6**	**2**	**5.1**	**2.1**	**7.2**	**7.5**	**3.1**	**3.0**
**SP_A=25**		**258**	**0.58**	**0.05**	**0.42**	**71.0**	**8.28**	**82.8**	**0**	**2.6**	**2.8**	**7.3**	**7.3**	**3.1**	**3.0**
Upright	No	214	0.58	0.05	0.45	69.8	8.31	79.5	0	2.4	2.8	7.7	7.4	3.3	3.1
Reference	No	238	0.59	0.09	0.42	73.4	8.25	81.6	0	2.2	3.0	7.4	7.4	3.1	3.0
Slouched	No	322	0.56	0.01	0.41	69.9	8.27	87.4	0	3.4	2.6	6.9	7.1	2.9	3.0
**SP_A=15**		**316**	**0.52**	**0.01**	**0.41**	**64.9**	**8.58**	**92.7**	**1**	**3.6**	**2.5**	**7.5**	**7.8**	**3.2**	**3.0**
Upright	No	245	0.54	0.01	0.39	68.3	8.28	83.8	0	2.3	2.9	7.8	8.2	3.4	3.0
Reference	No	331	0.51	0.01	0.42	68.6	8.93	101.5	1	3.9	2.6	7.6	7.9	3.3	3.1
Slouched	Yes	371	0.52	0.00	0.44	57.7	8.54	92.9	1	4.5	2.0	7.0	7.4	2.9	3.1
**SP_A=5**		**394**	**0.47**	**0.02**	**0.36**	**47.5**	**7.83**	**78.1**	**4**	**9.0**	**0.9**	**6.7**	**7.4**	**3.0**	**3.1**
Upright	Yes	404	0.47	0.06	0.37	57.1	9.13	89.8	5	7.3	1.2	7.3	7.8	3.2	3.1
Reference	Yes	368	0.48	0.00	0.34	48.2	7.51	75.6	3	9.2	0.8	6.9	7.4	3.0	3.1
Slouched	Yes	411	0.47	0.01	0.36	37.1	6.86	69.1	5	10.7	0.6	5.9	6.8	2.7	3.1
**Pulse#2**															
**SB_A=22**		**374**	**0.55**	**0.37**	**0.50**	**48.0**	**8.13**	**55.8**	**0**	**3.7**	**1.6**	**9.5**	**8.3**	**4.2**	**3.0**
**SP_A=15**		**374**	**0.55**	**0.37**	**0.50**	**48.0**	**8.13**	**55.8**	**0**	**3.7**	**1.6**	**9.5**	**8.3**	**4.2**	**3.0**
Baseline	No	374	0.55	0.37	0.50	48.0	8.13	55.8	0	3.7	1.6	9.5	8.3	4.2	3.0
**SB_A=40**		**654**	**0.52**	**0.19**	**0.44**	**62.0**	**8.73**	**86.0**	**3**	**8.0**	**2.3**	**8.2**	**8.2**	**3.6**	**3.1**
**SP_A=25**		**781**	**0.61**	**0.28**	**0.48**	**74.9**	**8.87**	**90.3**	**1**	**4.1**	**3.5**	**8.6**	**8.1**	**3.6**	**3.0**
Upright	No	1355	0.62	0.56	0.51	77.0	9.15	88.0	0	2.9	3.7	9.0	8.3	3.9	3.1
Reference	No	433	0.62	0.20	0.50	77.5	9.12	88.7	1	4.3	3.8	8.8	8.3	3.7	3.0
Slouched	Yes	554	0.58	0.09	0.44	70.1	8.34	94.1	1	5.1	3.1	7.9	7.8	3.3	3.0
**SP_A=15**		**612**	**0.52**	**0.17**	**0.46**	**63.8**	**9.60**	**93.7**	**3**	**6.5**	**2.7**	**8.6**	**8.4**	**3.7**	**3.1**
Upright	No	518	0.55	0.15	0.49	68.5	9.63	89.1	4	4.4	3.3	9.3	8.7	4.0	3.0
Reference	Yes	615	0.52	0.22	0.45	67.3	9.74	98.0	1	5.4	2.8	8.7	8.6	3.8	3.0
Slouched	Yes	703	0.50	0.13	0.45	55.5	9.44	93.9	4	9.6	1.9	7.8	7.8	3.3	3.1
**SP_A=5**		**569**	**0.43**	**0.11**	**0.39**	**47.4**	**7.70**	**74.0**	**5**	**13.4**	**0.7**	**7.6**	**8.1**	**3.4**	**3.1**
Upright	Yes	742	0.44	0.29	0.39	51.4	8.30	75.7	5	13.8	0.9	8.1	8.4	3.5	3.1
Reference	Yes	745	0.44	0.03	0.38	48.4	7.95	72.5	6	15.4	0.6	7.65	7.96	3.31	3.1
Slouched	Yes	221	0.42	0.00	0.39	42.4	6.86	73.8	4	11.1	0.5	7.01	7.82	3.23	3.0
