# Supplementary material for: Effects of seat pan and pelvis angles on the occupant response in a reclined position during a frontal crash
Source: PLoS One. 2021 Sep 20;16(9):e0257292. doi: 10.1371/journal.pone.0257292 (PMC8452024; doi:10.1371/journal.pone.0257292)
Supplement: S4 Table — The forward excursion corresponds to the maximum excursion along the X-axis. The upright excursion corresponds to the one along the Z-axis while reaching the maximum forward excursion, for each body part; a negative value means a downward displacement. The gray rows give the average excursion for each seat configuration. SB_A: Seatback angle; SP_A: Seat pan angle. A Simulation stopped before reaching the maximum head and T1 forward excursions. B Simulation stopped before reaching the maximum head, T1, and pelvis forward excursions. (PDF) [file pone.0257292.s008.pdf]

	Submarining occurrence	Head excursion (mm) Forward	Head excursion (mm) Upright	T1 excursion (mm) Forward	T1 excursion (mm) Upright	Pelvis excursion (mm) Forward	Pelvis excursion (mm) Upright	Lap belt penetration (mm)
**Without airbag environment**								
**SB_A=40**								
**SP_A=25**								
Upright	No	746	-107	489	52	219	-5	-
Reference	No	703	-138	449	38	220	-2	-
Slouched [B]	No	507	-24	344	-4	246	-4	-
**SP_A=15**								
Upright	No	750	-180	491	7	269	-27	-
Reference	No	733	-207	475	-9	277	-25	-
Slouched	Yes	724	-211	469	-22	301	2	75
**SP_A=5**								
Upright	Yes	771	-327	517	-114	371	-56	80
Reference [B]	Yes	564	119	389	-88	352	-53	77
Slouched	Yes	653	-160	445	-97	385	-37	88
**Lap belt anchorages fixed environment**								
**SB_A=40**								
**SP_A=25**								
Upright	No	658	-18	434	70	216	-6	-
Reference	No	631	-40	407	52	221	-1	-
Slouched	No	619	-57	401	20	243	-3	-
**SP_A=5**								
Upright	Yes	596	-129	428	-98	370	-64	76
Reference	Yes	599	-145	438	-111	385	-50	87
Slouched	Yes	594	-123	425	-93	384	-37	86
**Without airbag and lap belt anchorages fixed environment**								
**SB_A=40**								
**SP_A=25**								
Upright	No	743	-110	486	50	212	-7	-
Reference	No	701	-142	447	35	218	-3	-
Slouched [A]	No	665	-80	427	15	243	-4	-
**SP_A=5**								
Upright	Yes	785	-336	529	-113	374	-58	81
Reference	Yes	688	-193	467	-106	385	-50	87
Slouched	Yes	646	-150	443	-94	385	-39	87
